# Targeting the RSV and hMPV L Protein: Cryo-EM and Structure-Based Approaches to Antiviral Drug Discovery

**DOI:** 10.3390/biom16071020

**Published:** 2026-07-13

**Authors:** Yoon Ho Park, Rana Kim, Kun-Ho Song, Hyun Suk Jung

**Affiliations:** 1 Department of Biochemistry, College of Natural Sciences, Kangwon National University, Chuncheon 24341, Republic of Korearana1219@kangwon.ac.kr (R.K.); 2 University-Industry Cooperation Foundation, Kangwon National University, Chuncheon 24341, Republic of Korea

**Keywords:** human metapneumovirus, RNA-dependent RNA polymerase, Cryo-electron microscopy, antiviral drug design, L protein, polymerase inhibitors, Pneumoviridae

## Abstract

Respiratory syncytial virus (RSV) and human metapneumovirus (hMPV), members of the family Pneumoviridae, represent a foremost global cause of acute lower respiratory tract infection in infants, young children, the elderly, and immunocompromised individuals. Despite the recent approval of preventive vaccines and monoclonal antibody prophylactics targeting the viral fusion protein, no widely adopted, RSV-specific direct-acting antiviral is currently approved for routine post-infection treatment. The large (L) protein of the viral RNA polymerase complex, which catalyzes genome replication and mRNA transcription in concert with its obligate cofactor, the phosphoprotein (P), constitutes an ideal drug target owing to its essential and multifunctional enzymatic activities and its absence from host cells. Over the past decade, Cryo-electron microscopy (Cryo-EM) has yielded a series of landmark structures of Pneumoviridae L–P complexes, including apo forms of RSV (at 3.2–3.67 Å) and hMPV (at 3.7 Å) polymerases, among the first promoter-bound non-segmented negative-sense (nsNSV) RNA virus polymerase structures (at 3.40–3.41 Å), and inhibitor-bound complexes that illuminate the molecular basis of non-nucleoside inhibitor (NNI) action at sub-nanomolar potency. This review synthesizes the structural biology of Pneumoviridae RNA polymerases from a chronological and mechanistic perspective, compares RSV and hMPV L protein active sites at near-atomic resolution, and critically evaluates how structural insights are being translated into next-generation antiviral drug candidates, including nucleoside analog inhibitors, allosteric non-nucleoside inhibitors, and emerging candidates at various stages of preclinical and clinical investigation.

## 1. Introduction

Respiratory syncytial virus (RSV) and human metapneumovirus (hMPV) are enveloped, non-segmented, negative-sense (nsNSV) RNA viruses classified within the family Pneumoviridae, order Mononegavirales [1]. RSV, first isolated in 1956, and hMPV, discovered in 2001 [2,3], are the two most clinically significant members of this family and together account for a disproportionate share of the global acute lower respiratory infection (ALRI) burden in pediatric and vulnerable adult populations.

The epidemiological impact of RSV is substantial. According to World Health Organization estimates, RSV causes over 3.6 million hospitalizations and approximately 100,000 deaths annually in children under five years of age worldwide. Global burden estimates place RSV-associated ALRI among children at approximately 33.1 million episodes per year, with the overwhelming majority of severe outcomes and deaths occurring in low- and middle-income countries [4,5]. In the United States alone, RSV-associated pediatric hospitalizations incur direct medical costs estimated at $1.6 billion annually. The burden of hMPV, while less extensively quantified, is comparably severe; global estimates for 2018 identified approximately 14.2 million hMPV-associated ALRI cases and 643,000 hospitalizations in children under five, with the highest rates among infants under six months of age [5]. The clinical spectrum of both pathogens includes bronchiolitis, pneumonia, and croup, and RSV additionally constitutes a significant cause of severe respiratory disease in the elderly and in immunocompromised adults [1]. RSV circulates as two antigenically distinct subgroups (RSV-A and RSV-B), and hMPV is classified into two main lineages (A and B), each subdivided further; these genetic and antigenic differences are relevant to surveillance, cross-protection, and potentially to inhibitor susceptibility profiles [1,6].

The recent approval of RSV vaccines (Arexvy, Abrysvo) and messenger RNA-based vaccines (mResvia) targeting the prefusion conformation of the fusion (F) protein, together with the long-acting monoclonal antibody nirsevimab (Beyfortus) for infant prophylaxis, mark a milestone in RSV prevention. Nevertheless, all currently licensed interventions are preventive rather than therapeutic. No widely adopted, RSV-specific direct-acting antiviral is currently approved for routine treatment of established RSV or hMPV infection, although ribavirin retains historical regulatory approval and sees limited use in selected severe or immunocompromised RSV cases; its clinical benefit remains debated [1]. This represents an unmet medical need, particularly for hospitalized infants, immunocompromised patients, and the elderly in whom infection can be protracted and life-threatening.

Beyond these preventive measures, the F protein itself remains the most clinically validated RSV drug target. Structural definition of its metastable prefusion conformation [7] enabled the prefusion-stabilized vaccines and monoclonal antibodies noted above, as well as small-molecule fusion inhibitors that block viral entry; ziresovir, an orally administered F-protein inhibitor, recently demonstrated efficacy in hospitalized infants in a Phase 3 trial [8]. Because these fusion-directed agents act only at the entry step and are best suited to prophylaxis or early intervention, targeting the L polymerase, which drives all viral RNA synthesis throughout infection, offers a complementary, direct-acting antiviral strategy.

The RNA-dependent RNA polymerase (RdRp) machinery of Pneumoviridae viruses is a compelling target for antiviral drug development. The large (L) protein, the catalytic subunit of the polymerase, is a multifunctional enzyme solely responsible for all steps of viral RNA synthesis: genome replication, mRNA transcription, mRNA capping via a GDP polyribonucleotidyltransferase (PRNTase) mechanism, and methylation of the cap structure [9,10]. The L protein has no functional homologue among cellular enzymes for its RdRp and PRNTase activities, though its methyltransferase (MTase) domain shares structural similarity with host class I methyltransferases, a distinction with important implications for inhibitor selectivity that is discussed in Section 3. The phosphoprotein (P), the obligate cofactor of L, also plays central regulatory roles; its extensive phosphorylation modulates interactions with L, with the nucleoprotein (N), and with the transcription antiterminator M2-1 [6,11].

Over the past decade, Cryo-EM has transformed our structural understanding of the Pneumoviridae polymerase complex. From the first complete structures of RSV and hMPV L–P complexes in 2019–2020 to the landmark promoter-bound and inhibitor-bound structures resolved at 2.39–3.41 Å in 2023–2024, the field has established a detailed framework for both the mechanisms of RNA synthesis and the structural basis of small-molecule inhibition. This review synthesizes these advances from a chronological and mechanistic perspective, with particular emphasis on what Cryo-EM structures have directly established for Pneumoviridae and what remains inferred by analogy from other Mononegavirales systems. We describe the essential biological context of RNA synthesis, including the roles of accessory factors M2-1 and M2-2 and the viral inclusion bodies in which replication occurs, and critically evaluate the structure-guided antiviral pipeline, explicitly distinguishing peer-reviewed clinical evidence from preclinical findings.

This review is distinguished from prior structural reviews of Pneumoviridae polymerases by its emphasis on the 2023–2024 structural wave of promoter-bound and inhibitor-bound structures. It differs from Bonneux et al. 2025 [12], which focuses on AlphaFold modeling of unresolved domains, by centering on experimental Cryo-EM structures; from Cao and Liang 2021 [13], which covered only apo structures; and from Wolf et al. 2024 [14], which provides a broader paramyxo and pneumovirus scope compared with the Pneumoviridae-specific depth offered here.

## 2. The Pneumoviridae RNA Polymerase Complex

**Figure 1:**
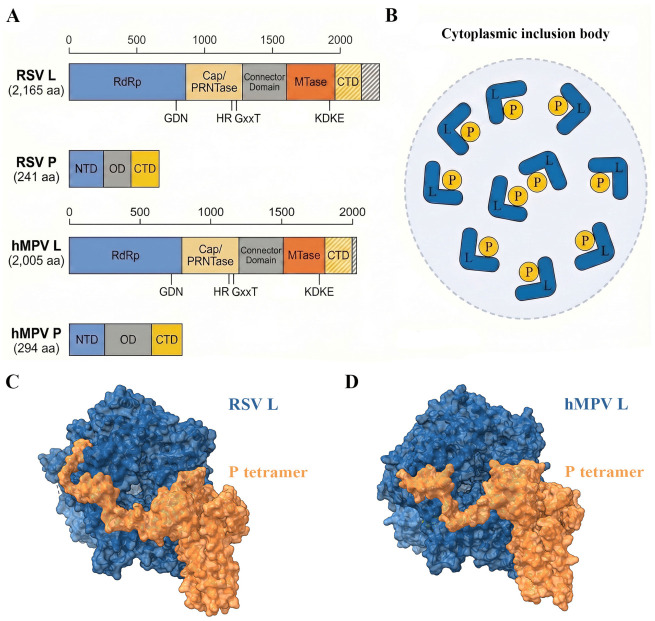
Domain architecture, three-dimensional structure, and cellular context of Pneumoviridae RNA polymerases. (**A**) Linear domain maps of RSV L (2165 aa) and hMPV L (2005 aa) showing the five structural domains (RdRp, Cap/PRNTase, connector domain (CD), MTase, and CTD) with key catalytic motif positions indicated (RSV L numbering: GDN 810–812; HR His1338; GxxT; KDKE), together with the P protein maps of RSV P (241 aa) and hMPV P (294 aa) showing the NTD, OD, and CTD regions. (**B**) Schematic of L–P complexes within a cytoplasmic inclusion body (membraneless compartment). (**C**) Surface representation of the RSV L–P complex (PDB: 6UEN), with the L protein in blue and the tetrameric phosphoprotein (P) in orange. (**D**) hMPV L–P complex (PDB: 6U5O) shown in the same color scheme and a comparable orientation, illustrating the conserved architecture of the Pneumoviridae L–P complex.

L protein domain architecture: The L proteins of RSV (2165 amino acids; ~250 kDa) and hMPV (2005 amino acids; ~228 kDa) are single polypeptide chains that fold into five structurally and functionally distinct domains, arranged from N- to C-terminus: the RNA-dependent RNA polymerase (RdRp) domain, the capping/polyribonucleotidyltransferase (Cap/PRNTase) domain, the connector domain (CD), the methyltransferase (MTase) domain, and the C-terminal domain (CTD) [11,13,15] (Figure 1A,B). This five-domain architecture is conserved across the Mononegavirales and was first delineated at the sequence level through the identification of six conserved regions (CR I–VI) across rhabdoviral, paramyxoviral, and pneumoviral L proteins [16].

The RdRp domain (approximately residues 1–940 in RSV L) adopts the canonical right-hand polymerase fold comprising palm, fingers, and thumb subdomains, with the palm housing the catalytic GDN motif (Gly810-Asp811-Asn812 in RSV L) essential for nucleotidyl transfer [17]. Conserved sequence motifs A through F within the RdRp domain coordinate two divalent metal ions (typically Mg^2+^ or Mn^2+^) required for the two-metal-ion mechanism of nucleotide polymerization [15]. The Cap/PRNTase domain is structurally unique to nsNSV RNA viruses and performs a two-step capping reaction. The conserved His residue of the HR motif (His1338 in RSV L) forms a covalent phosphoamide intermediate with the 5′ end of the nascent mRNA, and this RNA is then transferred to GDP via the GxxT motif to produce the cap-0 structure (GpppN) [10,11]. The Cap domain also harbors the priming loop, a critically dynamic structural element involved in de novo initiation of RNA synthesis, whose conformational state differs markedly among nsNSV polymerase structures. The connector domain (CD) is the least sequence-conserved domain among nsNSV L proteins (the least conserved region between RSV and hMPV) and serves as a structural bridge linking the catalytic core to the peripheral MTase–CTD module [11,13]. The MTase domain belongs to the class I methyltransferase superfamily and catalyzes cap methylation using S-adenosylmethionine (SAM) as the methyl donor; its catalytic tetrad KDKE is strictly conserved across Mononegavirales [15]. By analogy with the resolved VSV and PIV5 CTD–MTase modules, the CTD is thought to form a structurally integrated unit with the MTase and contributes additional SAM-cofactor contacts and structural integrity to the MTase active site.

P protein and its regulatory roles: The P protein is the essential cofactor of L and the molecular hub of the Pneumoviridae replication machinery. RSV P (241 amino acids; 27 kDa) and hMPV P (294 amino acids; 32.5 kDa) differ in size but both form stable homotetramers in solution [1,18]. The name “phosphoprotein” reflects constitutive and dynamic phosphorylation that profoundly modulates P’s activities. RSV P is phosphorylated at Ser116, Ser117, Ser119 (central cluster) and Ser232, Ser237 (C-terminal cluster) by cellular casein kinase II [19], and this phosphorylation is required for efficient virus replication in vivo. P is organized into three functional regions: an N-terminal domain (NTD) that binds nascent N protein (N^0^); an oligomerization domain (OD) that drives tetramer formation; and a C-terminal domain (CTD) that engages the L protein surface [11,18]. The N^0^-binding function of the P NTD is essential for genome replication, ensuring a continuous supply of unassembled N subunits for co-replicational encapsidation of the nascent antigenome and genome strands. P also contains the binding site for the M2-1 transcription antiterminator (residues ~90–110 of RSV P) [20,21]. The phosphorylation state of P influences its interactions with L, with N–RNA, and potentially with M2-1, though the structural consequences of individual phosphorylation events on the assembled L–P complex remain incompletely characterized.

L–P complex architecture from Cryo-EM: The structural elucidation of Pneumoviridae polymerases began in earnest with the 2019 publication by Gilman et al. of the RSV L–P complex at 3.2 Å resolution (PDB: 6PZK) [22]. This study resolved the RdRp and Cap domains of RSV L bound to the P OD and P CTD, establishing the overall ring-shaped architecture of the RSV polymerase core. The structure confirmed conservation of the active site GDN motif and the HR capping motif. The CD, MTase, and CTD of L, as well as the P NTD, were absent from the Cryo-EM density, indicating significant conformational flexibility of these peripheral elements. The priming loop (residues 1265–1282 in RSV L) was displaced by approximately 37.2 Å and rotated by ~146° compared to the VSV priming loop position [9], suggesting, by analogy with VSV structural assignments, that this conformation is consistent with an elongation-compatible rather than pre-initiation state [22].

Shortly thereafter, Cao et al. reported a second RSV L–P structure at 3.67 Å resolution (PDB: 6UEN), independently confirming the overall architecture and providing further detail on the L–P interface [17]. Both P OD and P CTD chains were found to engage the RSV L RdRp domain through distinct interaction surfaces, with one P CTD chain extending to contact the palm motif near the putative NTP entry channel.

Pan et al. reported the first structure of the hMPV L–P complex at 3.7 Å resolution (PDB: 6U5O) [18]. The hMPV polymerase core adopts a core fold essentially identical to that of RSV L, with an RMSD of 1.34 Å over 1501 aligned Cα atoms [18]. A distinguishing feature was the priming loop conformation: in hMPV L, the priming loop is fully retracted into a cavity within the Cap domain [18]. The P tetramer in the hMPV structure wraps around the L core in a tentacular manner, with two P CTD chains extending across the RdRp surface [18]. As in all RSV structures, the CD and MTase–CTD modules were absent from the structure.

Together, these three studies established the conserved ring-shaped architecture of Pneumoviridae L–P complexes (Figure 1C,D), defined the primary L–P interaction surfaces, and provided initial structural frameworks for understanding nucleotide polymerization and cap synthesis, although the persistent absence of the CD–MTase–CTD module left fundamental questions about cap methylation and the transcription–replication switch unanswered. A parallel X-ray crystal structure of the hMPV MTase–CTD (CR-VI+ domain) at 3.26 Å resolution (PDB: 4UCJ), reported in 2015 by Paesen et al. [23], had already provided a high-resolution view of an nsNSV MTase domain and defined the SAM-binding groove and KDKE active site architecture, and this structure remains critical for informing MTase inhibitor design.

The Pneumoviridae structural advances have occurred alongside a broader revolution in nsNSV polymerase structural biology (Table 1). The founding structure, VSV L at 3.8 Å (PDB: 5A22) in 2015 [9], first established the five-domain ring architecture. Subsequent structures of rabies virus (RABV) L–P [24] and VSV L–P [25] provided the first view of P-induced ordering of the CD and MTase–CTD module. PIV5 L–P [26] demonstrated a unique MTase–CTD conformation inferred to be relevant to transcription regulation. Ebola virus L–VP35 [27] and the Nipah virus L–P structure at 3.19 Å (PDB: 9DKU) [28] provided high-resolution views of filoviral and henipaviral polymerases, respectively, and confirmed that the PRNTase allosteric pocket exploited by JNJ-8003 and MRK-1 is a conserved feature across nsNSV polymerases, though natural sequence divergence at this site can confer differential inhibitor susceptibility [28].

Read as a whole, Table 1 captures the maturation of the field along two axes. Resolution has improved steadily from the founding VSV structure at 3.8 Å (2015) to 2.39 Å for the RSV MRK-1 complex (2023), reflecting the broader Cryo-EM resolution revolution, while the functional states captured have progressed from apo and P-bound cores (2015–2020) to promoter-bound and inhibitor-bound complexes (2023–2024). Notably, Pneumoviridae are the only family represented by both promoter-bound (8SNX, 8SNY) and inhibitor-bound (8FPI, 8FPJ, 8FU3) high-resolution structures, whereas the Rhabdoviridae, Paramyxoviridae, and Filoviridae entries remain limited to apo or cofactor-bound states. RSV and hMPV are therefore the most completely characterized and most extensively drug-validated nsNSV polymerases to date; the conservation of the PRNTase allosteric pocket across these families suggests broad-spectrum potential, but the current absence of inhibitor-bound structures outside Pneumoviridae remains a clear gap for cross-family antiviral development.

M2-1, M2-2, and the cellular context of RNA synthesis. A comprehensive understanding of Pneumoviridae polymerase function requires situating the L–P core within the broader replication machinery that operates in infected cells. The purified L–P complex, while indispensable as a structural model, represents only one component of the complete transcription and replication apparatus.

The M2-1 protein (194 amino acids in RSV) is an essential transcription antiterminator without which infectious RSV cannot be recovered from cDNA [32]. In its absence, the viral polymerase terminates prematurely within long viral mRNAs and fails to reinitiate at downstream gene-start signals [32]. Tanner et al. determined the crystal structure of full-length RSV M2-1 at 2.52 Å resolution (PDB: 4C3B), revealing that M2-1 forms a disk-like homotetramer stabilized by a central four-helix bundle [21]. Each monomer comprises an N-terminal zinc-binding domain (ZBD; residues 7–25) essential for antitermination activity, a tetramerization helix (residues 32–49), and a globular core domain (residues 69–172) responsible for RNA binding [21]. The interaction of M2-1 with P protein (via P residues ~90–110) was characterized by Blondot et al., revealing that a P peptide forms an alpha-helix within a cleft of the M2-1 core domain [20]. This M2-1/P interaction is relatively weak, and high-affinity RNA can displace M2-1 from P, providing the structural basis for a model in which M2-1 is handed from P to the nascent RNA during the elongation-to-termination decision [20]. Two serine residues (Ser58 and Ser61) are dynamically phosphorylated and modulate M2-1 antitermination activity [21]. M2-1 is specific to transcription and has no role in genome replication, providing a potential therapeutic angle distinct from the L protein catalytic sites.

The M2-2 protein (90 amino acids in RSV), encoded by a second overlapping ORF within the M2 mRNA, acts as a regulatory switch governing the balance between transcription and replication. Bermingham and Collins demonstrated that viruses lacking M2-2 synthesize 2–4-fold more mRNA and 3–4-fold less genomic RNA than wild-type virus and replicate approximately 1000-fold less efficiently in the initial days of infection [33]. As M2-2 accumulates during infection, transcription is progressively downregulated and genome replication is upregulated [33]. The molecular mechanism by which M2-2 communicates with the polymerase complex remains an open question; a high-resolution structure of M2-2 in complex with any component of the RNA synthesis machinery is not yet available.

**Figure 2:**
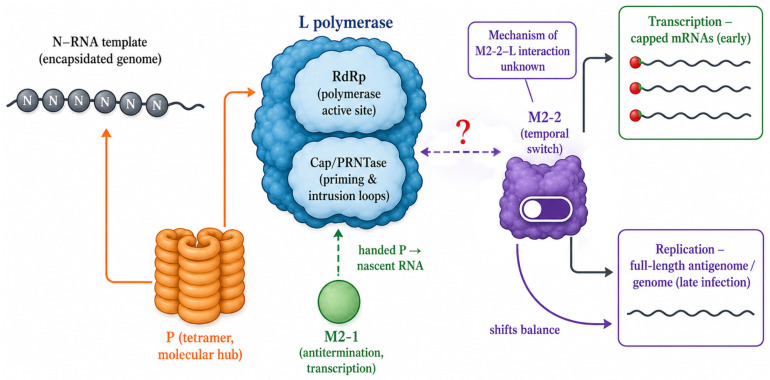
Schematic model of the Pneumoviridae L–P–M2 RNA-synthesis machine and the transcription-to-replication switch. The N-encapsidated RNA template (gray) is depicted as a single-stranded RNA coated with nucleoprotein (N). The tetrameric phosphoprotein (P, orange) bridges the L polymerase (blue; RdRp and Cap/PRNTase domains indicated) and the template. M2-1 (green), the transcription antitermination factor, and M2-2 (purple), the temporal switch that shifts synthesis from transcription toward replication, act on the RNA-synthesis machinery; the green dashed arrow (“handed P → nascent RNA”) denotes the proposed transfer of M2-1 and the purple arrow (“shifts balance”) the direction of the M2-2 effect. The right-hand boxes represent the products of the polymerase: capped mRNAs (transcription, early infection; green) and full-length antigenome and genome (replication, late infection; purple). Solid arrows indicate established interactions or flow, and dashed arrows indicate interactions that are proposed or not yet structurally resolved; the dashed purple arrow with a red question mark marks the M2-2–polymerase interaction, for which no experimental structure is currently available.

Taken together, the L–P core and the M2 proteins constitute a single regulated machine that partitions the polymerase between transcription and replication. On the encapsidated N-RNA template, P couples L to the nucleoprotein and, early in infection, recruits M2-1 to sustain processive, full-length mRNA synthesis by suppressing premature termination [32]; as M2-2 accumulates later, it shifts the balance from transcription toward genome replication [33]. P thus serves as the physical hub linking L to the template and to M2-1, while M2-2 provides the temporal switch that converts the machine from a transcriptase into a replicase. The molecular basis of this switch remains undefined, however: no structure of M2-2 bound to any component of the polymerase has been determined, so a definitive interaction model awaits future Cryo-EM and in situ studies (Figure 2).

In RSV- and hMPV-infected cells, viral RNA synthesis is concentrated within cytoplasmic membraneless compartments known as inclusion bodies (IBs) [34,35]. These structures exhibit liquid-like properties consistent with phase separation driven by multivalent interactions among N, P, and M2-1 [34]. Within the IB, the L–P polymerase engages N-encapsidated RNA, not naked RNA, as its template [11,35]. This distinction means that structural studies of purified L–P complexes on synthetic RNA templates illuminate the core enzymatic machinery but cannot fully recapitulate the dynamics of template engagement, antitermination, or the transcription-to-replication switch as they occur in the native cellular context. Importantly, these regulatory roles of IBs are inferred from live-cell imaging and in vitro reconstitution rather than from in situ structural data; direct structural visualization of the active polymerase within IBs is not yet available and remains a principal objective for the Cryo-electron tomography approaches discussed below.

## 3. Structural Mechanisms of RNA Synthesis and Cap Formation

RdRp catalysis and promoter recognition. The promoter-bound RSV polymerase structures reported by Cao et al. in 2024—the L–P complex with the Le10 genomic leader promoter (PDB: 8SNX; 3.40 Å) and with the TrC10 trailer complementary promoter (PDB: 8SNY; 3.41 Å)—are among the first nsNSV viral RdRp–RNA promoter complexes solved by Cryo-EM and provide the most direct view of RSV polymerase template engagement available to date [31].

The critical structural feature revealed by these structures is the ordering of the “supporting helix” (residues 666–676) and “supporting loop” (residues 656–665), which are disordered in all apo L–P structures but become fully resolved upon promoter RNA binding, forming one wall of the template entry channel and making direct contacts with the 3′ end of the promoter RNA [31]. Promoter binding is also associated with a small inward shift in the Cap domain (~1.8 Å) relative to the apo state, generating a more compact catalytic pocket [31]. These observations are consistent with a model in which template engagement drives a conformational tightening of the enzyme around its substrate, though caution is warranted in assigning these static structures to discrete physiological states in the absence of direct time-resolved functional validation.

The Le10 and TrC10 promoters bind in an identical channel but position their nucleotides differently at the catalytic site: in the Le10-bound structure, the +3 nucleotide occupies the active site, while in the TrC10-bound structure, the +1 nucleotide is positioned there [31]. This provides a direct structural basis for the well-characterized ability of RSV polymerase to initiate RNA synthesis at two distinct positions on its promoter, a property previously characterized only biochemically [6,36]. The promoter-bound structures suggest that the supporting helix and loop stabilize the incoming template strand and position the initiating nucleotide within reach of the catalytic metals, but direct visualization of a covalent initiation intermediate has not yet been achieved [31].

No elongating Pneumoviridae polymerase complex containing a full template–product RNA duplex has been solved. Consequently, the product exit channel geometry, the conformational changes in the palm and fingers subdomains accompanying processive nucleotide incorporation, and the exact path of the nascent RNA through this channel remain to be established experimentally for RSV and hMPV.

PRNTase domain: priming, capping, and allosteric inhibition. The Cap/PRNTase domain has emerged as the most structurally validated drug target on the Pneumoviridae polymerase, owing to the convergence of Cryo-EM inhibitor-bound structures, biochemical mechanism-of-action studies, and resistance profiling.

The priming loop (residues 1265–1282 in RSV L) occupies different positions across nsNSV polymerase structures. In VSV L, it partially occludes the central RNA-binding cavity, consistent with a pre-initiation state. In RSV apo (PDB: 6UEN) [17] and promoter-bound structures [31], the priming loop is shifted away from the central cavity. In hMPV L (PDB: 6U5O) [18], it is fully retracted into a Cap domain cavity. These positions support a model of functionally distinct conformational states across the initiation-to-elongation transition, though whether they represent true functional states or partly reflect differences in sample preparation remains unresolved, and their precise assignment to discrete catalytic steps awaits elongation complex structures not yet available for Pneumoviridae [11,15].

**Figure 3:**
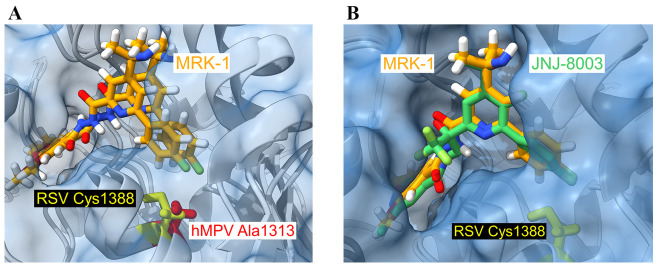
Structural basis of non-nucleoside inhibitor binding at the PRNTase-domain allosteric pocket. (**A**) Close-up of the allosteric pocket shown as the molecular surface of the RSV MRK-1 structure (PDB: 8FPI), onto which the MRK-1-bound hMPV structure (PDB: 8FPJ) is superimposed; two MRK-1 molecules, one from each structure and near-completely overlapping, are therefore displayed. The single pocket-lining difference, RSV Cys1388 (yellow) versus hMPV Ala1313 (red), is highlighted and provides the residue-level basis for the ~90-fold difference in MRK-1 EC_50_ between RSV and hMPV [29]. (**B**) The same pocket, shown as the surface of the RSV structure (PDB: 8FPI), comparing the binding modes of MRK-1 (gold; PDB: 8FPI) and JNJ-8003 (green; PDB: 8FU3); both inhibitors occupy the Cys1388-containing pocket but engage it with different geometry, consistent with the induced-fit mode of JNJ-8003 [29,30]. Ligand carbons are colored by molecule (MRK-1, gold; JNJ-8003, green) and heteroatoms by element (N, blue; O, red; H, white); highlighted pocket-lining residues are shown in yellow (RSV Cys1388) and red (hMPV Ala1313).

Two pivotal Cryo-EM studies published in 2023 established the PRNTase domain allosteric pocket as a structurally defined and druggable target (Figure 3). Kleiner et al. reported the structure of MRK-1 bound to RSV L–P at 2.39 Å (PDB: 8FPI) and to hMPV L–P at 2.74 Å (PDB: 8FPJ) [29]. These near-atomic-resolution structures provided the first direct visualization of a non-nucleoside inhibitor bound simultaneously to both RSV and hMPV polymerases in the same study, confirming conservation of the allosteric pocket and revealing that a single residue difference, RSV Cys1388 versus hMPV Ala1313, accounts in part for the approximately 90-fold difference in MRK-1 EC_50_ between the two viruses (2.1 nM for RSV versus 185 nM for hMPV) (Figure 3A) [29]. Competition binding assays established that the MRK-1 pocket is distinct from those targeted by AZ-27, BI-D, or PC786 [29].

Contemporaneously, Yu et al. characterized JNJ-8003 at 2.9 Å (PDB: 8FU3), revealing an induced-fit binding mode at the same PRNTase pocket [30]. JNJ-8003 demonstrated sub-nanomolar inhibitory potency (EC_50_ = 0.78 nM; IC_50_ = 0.67 nM) and showed in vivo activity in murine and neonatal lamb models [30]. The structure showed that JNJ-8003 binding modulates the functional interplay between the Cap and RdRp domains and is proposed to trap the priming loop and intrusion loop in non-productive conformations [30] (Figure 3B).

Any inhibitor that stabilizes the Cap domain in a non-productive conformation at the functionally essential priming loop–intrusion loop interface will impede RNA synthesis, regardless of its precise chemical contacts [11,29,30]. Wolf et al. 2024 have further articulated this concept by framing small-molecule inhibitors as conformational probes that can stabilize transitory polymerase states [14]. For NNI resistance, mutations identified from in vitro selection for both MRK-1 and JNJ-8003 map to Cap domain residues near the priming loop and at the periphery of the binding pocket [29,30,37]. More broadly, because such allosteric inhibitors act by shifting the conformational equilibrium of the polymerase toward inhibitor-trapped, non-productive states rather than by engaging a single rigid pocket, their effective affinity is intrinsically coupled to the conformational state of the enzyme; the static cryo-EM structures capture the endpoints of this equilibrium, whereas the dynamic transitions between initiation, priming, and elongation states that gate inhibitor access remain to be characterized.

Cap methylation: the RSV N7-first pathway. The MTase domain catalyzes the sequential methylation of the cap-0 (GpppN) structure. In VSV, biochemical studies established that 2′-O-methylation precedes N7-methylation [38]. However, Sutto-Ortiz et al. demonstrated in 2021 that the RSV MTase-CTD catalyzes cap methylation with a kinetic preference for N7-methylation as the first reaction, generating m7GpppN; only subsequently does 2′-O-methylation occur to produce the mature m7GpppNm (cap-1) structure [39]. This RSV-specific N7-first order contrasts with the canonical VSV 2′-O-first order [38] and has significant implications for drug design. The hMPV MTase-CTD crystal structure at 3.26 Å (PDB: 4UCJ) [23] provides the best available structural model for the Pneumoviridae MTase active site, but given only ~32% sequence identity between the RSV and hMPV MTase domains, differences in peripheral surfaces may have pharmacological relevance [12].

The connector domain and C-terminal module. In VSV L [9] and PIV5 L–P [26], specific P-derived contacts lock the CD and MTase–CTD in defined orientations. In all Pneumoviridae structures, the CD and downstream domains are consistently absent from Cryo-EM density, indicating high conformational flexibility [11,17,18]. Based on structural homology with VSV and PIV5 systems, the CD is presumed to enable regulated positioning of the MTase–CTD module relative to the catalytic core during transcription and replication [11,15], but direct evidence in Pneumoviridae awaits experimental determination.

## 4. Drug Target Conservation and Antiviral Development

RSV–hMPV active site conservation. RSV and hMPV L proteins share approximately 48% overall amino acid identity [18], with conservation markedly higher in the catalytic RdRp and capping domains than in the connector and methyltransferase–CTD regions [12,15]. At the level of catalytic motifs, conservation is essentially absolute: the GDN triad, the HR and GxxT motifs, and the KDKE tetrad are fully conserved between RSV and hMPV L proteins [29,30]. Structural superimposition of the RSV and hMPV polymerase cores reveals near-identical active-site architecture (overall core RMSD of 1.34 Å over 1501 aligned Cα atoms) [18], indicating that the catalytic regions are under stronger evolutionary constraint than the peripheral elements.

The MRK-1/JNJ-8003 binding pocket on the Cap domain is formed by residues from conserved motifs B, D, and E. Four of five residues making direct side-chain contacts with the inhibitor are identical between RSV and hMPV, while RSV Cys1388 is replaced by Ala1313 in hMPV [29]. This single substitution accounts in part for the ~90-fold potency difference. At least five distinct druggable sites can be identified on the Pneumoviridae L–P complex: (i) the RdRp active site; (ii) the Cap/PRNTase catalytic site; (iii) the allosteric Cap domain pocket; (iv) the MTase SAM-binding groove; and (v) the L–P protein–protein interaction interface [11,12,37].

Nucleoside analog inhibitors: Nucleoside analogs targeting the RdRp active site function as obligate or delayed chain terminators [37,40]. The most clinically advanced nucleoside inhibitor for RSV was lumicitabine (ALS-8176; JNJ-64041575), a 4′-chloromethyl-2′-fluoro-2′-deoxycytidine prodrug. In a Phase IIa human RSV challenge study in healthy adults, lumicitabine resulted in 73–88% reduction in RSV exposure relative to placebo [41]. Subsequent Phase IIb studies in hospitalized infants revealed dose-limiting, reversible neutropenia, a host toxicity distinct from antiviral activity, and lumicitabine was discontinued [42]. In vitro resistance selection identified the “QUAD” substitutions (M628L, A789V, L795I, and I796V) in the L RdRp domain, which reduce incorporation of the active metabolite (ALS-8112-triphosphate) [11,37].

4′-Fluorouridine (EIDD-2749) is a broad-spectrum ribonucleoside analog with a distinctive delayed chain-termination mechanism. Sourimant et al. demonstrated that 4′-FlU-TP is incorporated by both RSV and SARS-CoV-2 polymerases in place of UTP [43]. GS-7682, a phosphoramidate prodrug of the 4′-cyano C-nucleoside GS-646089, showed potent broad-spectrum activity against RSV (EC_50_ = 3–46 nM) and hMPV (EC_50_ = 210 nM), and significantly reduced RSV viral loads in African green monkeys [44]. GS-7682 remains in preclinical development.

Non-nucleoside allosteric inhibitors. Beyond JNJ-8003 and MRK-1, several other NNI chemotypes target the RSV Cap/PRNTase domain. AZ-27 and the related YM-53403 inhibit RSV replication with micromolar potency, with resistance mapping to the linker region between the CD and MTase domains (Y1631H) [6,37]. BI-D targets the Cap domain with nanomolar potency [29,37]. PC786, an inhaled non-nucleoside RSV L-protein polymerase inhibitor developed by Pulmocide with low nanomolar potency, maps to a partially overlapping site [37]; its clinical development was subsequently discontinued during Phase II [45].

Emerging clinical candidates and combination strategies: S-337395 (Shionogi/UBE) is an investigational oral small-molecule RSV L protein inhibitor. In January 2025, Shionogi disclosed results from a randomized, placebo-controlled Phase 2 human challenge trial (company press release; peer-reviewed publication awaited): the highest dose achieved an approximately 89% reduction in viral load compared to placebo, with no serious adverse events. S-337395 has received Fast Track designation from the US FDA. EDP-323 (Enanta) is a first-in-class oral non-nucleoside RSV L protein inhibitor; Phase 1 data showed favorable safety and pharmacokinetics supporting once-daily oral dosing [46], and Phase 2a human challenge results presented at IDWeek 2025 reported 85–87% reductions in viral load AUC versus placebo. Both data sets were obtained in healthy adult volunteers under controlled inoculation, conditions far removed from natural severe RSV disease. The identification of multiple structurally distinct druggable sites raises the possibility of combination therapy; preliminary evidence for synergistic activity between NIs and NNIs against RSV has been reported [37]. This review focuses on inhibitors directed at the L polymerase; small molecules targeting other RSV proteins, such as the nucleoprotein-directed replication inhibitor EDP-938 [47] and the F-protein fusion inhibitors noted above, act through distinct mechanisms and lie outside the scope of this L-protein-focused review.

Cross-viral broad-spectrum strategies. Because the catalytic core of the L protein is the most strongly conserved region across Pneumoviridae, active-site-directed inhibitors provide the clearest route to broad-spectrum activity against both RSV and hMPV. The near-identical RSV and hMPV active-site architecture described above allows a single chemical scaffold to engage both enzymes: the non-nucleoside inhibitor MRK-1 binds the conserved PRNTase pocket of both viruses, although the Cys1388/Ala1313 difference lowers its hMPV potency approximately 90-fold, illustrating that even conserved pockets retain potency-determining residues that must be accommodated in pan-Pneumoviridae design [29]. Nucleoside analogs, which target the universally conserved RdRp catalytic center, achieve the broadest coverage: GS-7682 inhibits both RSV and hMPV at nanomolar concentrations [44], and 4′-fluorouridine extends antiviral activity to unrelated respiratory RNA viruses including SARS-CoV-2 [43]. Together these examples define a coherent screening strategy for cross-viral inhibitors: prioritize the conserved RdRp and PRNTase catalytic sites over the divergent peripheral domains and use RSV–hMPV structural superposition to identify the few non-conserved pocket residues that govern differential potency.

## 5. Discussion

The past decade has witnessed a transformation in our structural understanding of Pneumoviridae RNA polymerases, driven principally by the resolution revolution in Cryo-EM. The promoter-bound structures moved the field beyond static apo snapshots, and the inhibitor-bound structures established that the PRNTase allosteric pocket is a structurally validated, high-resolution druggable site. The near-identical active-site architecture shared by RSV and hMPV, together with the single Cys1388/Ala1313 substitution that governs differential inhibitor potency, defines the structural boundary between conserved and divergent features of the two polymerases, while the RSV MTase preference for N7-first methylation, which distinguishes it from the VSV 2′-O-first paradigm, further illustrates that mechanistic detail rather than overall fold now separates these closely related enzymes.

These structural insights are already shaping ongoing small-molecule drug-discovery efforts. The inhibitor-bound PRNTase structures (8FPI, 8FPJ, 8FU3) provide direct templates for structure-based optimization of the MRK-1 and JNJ-8003 chemotypes and for rationalizing the Cys1388/Ala1313 selectivity gap, while the promoter-bound structures (8SNX, 8SNY) define the RdRp active-site geometry that nucleoside analogs and clinical-stage L inhibitors such as S-337395 and EDP-323 must engage. The RSV–hMPV active-site superposition further offers a residue-level blueprint for prioritizing broadly active scaffolds, so that these structures function not as descriptive endpoints but as an actionable platform for next-generation antiviral design.

Beyond these immediate applications, the structures carry broader implications for antiviral development. The validated allosteric pocket and the Cys1388/Ala1313 determinant provide both a structural rationale and a concrete design challenge for pan-Pneumoviridae inhibitor optimization, while the identification of at least five distinct druggable sites on the L–P complex opens a realistic path toward combination therapy that targets independent steps of RNA synthesis. Realizing this potential, however, depends on capturing the most functionally decisive states of the polymerase, which remain structurally undefined: no elongation complex, no full-length L protein with the CD, MTase, and CTD resolved, and no structure from the native replication context yet exists. We believe that Cryo-electron tomography (Cryo-ET) of infected cells, building on landmark in situ studies of other viral assemblies [48,49,50], together with computational approaches for conformational heterogeneity analysis, represents the most direct path to closing this gap and converting today’s structural insights into next-generation antivirals.

## 6. Conclusions

Cryo-EM has transformed the structural understanding of Pneumoviridae RNA polymerases, advancing the field from static views of the polymerase core to a mechanistic framework that links template engagement, cap synthesis, and small-molecule inhibition. The structurally validated PRNTase allosteric pocket, the single active-site determinant that distinguishes RSV from hMPV, and the distinctive cap-methylation order of the RSV enzyme together establish a clear structural foundation for antiviral design. Translating these insights into effective, broadly active therapeutics will require capturing the polymerase in its functionally decisive and native states, positioning structure-guided drug discovery as a central strategy against these clinically important respiratory pathogens.

## Figures and Tables

**Table 1:** Selected Cryo-EM and X-ray Structures of nsNSV Polymerases, with Emphasis on Pneumoviridae.

Virus	Family	Resolution (Å)	Domains Resolved	Ligand	PDB ID	Year
RSV M2-1	Pneumoviridae	2.52 (X-ray)	Full-length M2-1 tetramer	None	4C3B [21]	2014
VSV	Rhabdoviridae	3.8	RdRp, Cap, CD, MTase, CTD	None	5A22 [9]	2015
hMPV	Pneumoviridae	3.26 (X-ray)	MTase-CTD (CR-VI+)	None	4UCJ [23]	2015
RSV	Pneumoviridae	3.2	RdRp, Cap	P OD + CTD	6PZK [22]	2019
RSV	Pneumoviridae	3.67	RdRp, Cap	P OD + CTD	6UEN [17]	2020
hMPV	Pneumoviridae	3.7	RdRp, Cap	P tetramer	6U5O [18]	2020
RABV	Rhabdoviridae	3.3	RdRp, Cap, CD	P peptide	6UEB [24]	2020
VSV	Rhabdoviridae	3.0	RdRp, Cap, CD	P peptide	6U1X [25]	2020
PIV5	Paramyxoviridae	4.3	RdRp, Cap, CD, MTase, CTD	P	6V85 [26]	2020
EBOV	Filoviridae	3.3	RdRp, Cap	VP35	7Y4D [27]	2022
RSV	Pneumoviridae	2.39	RdRp, Cap	MRK-1, P	8FPI [29]	2023
hMPV	Pneumoviridae	2.74	RdRp, Cap	MRK-1, P	8FPJ [29]	2023
RSV	Pneumoviridae	2.9	RdRp, Cap	JNJ-8003, P	8FU3 [30]	2023
RSV	Pneumoviridae	3.40	RdRp, Cap	Le10 RNA, P	8SNX [31]	2024
RSV	Pneumoviridae	3.41	RdRp, Cap	TrC10 RNA, P	8SNY [31]	2024
NiV	Paramyxoviridae	3.19	RdRp, Cap	P	9DKU [28]	2024

## Data Availability

No new data were created or analyzed in this study. Data sharing is not applicable to this article.
